# Dual Regulation by Pairs of Cyclin-Dependent Protein Kinases and Histone Deacetylases Controls G1 Transcription in Budding Yeast

**DOI:** 10.1371/journal.pbio.1000188

**Published:** 2009-09-08

**Authors:** Dongqing Huang, Supipi Kaluarachchi, Dewald van Dyk, Helena Friesen, Richelle Sopko, Wei Ye, Nazareth Bastajian, Jason Moffat, Holly Sassi, Michael Costanzo, Brenda J. Andrews

**Affiliations:** 1 Banting and Best Department of Medical Research, University of Toronto, Toronto, Ontario, Canada; 2 Terrence Donnelly Centre for Cellular and Biomolecular Research, University of Toronto, Toronto, Ontario, Canada; Yale University, United States of America

## Abstract

START-dependent transcription in *Saccharomyces cerevisiae* is regulated by two transcription factors SBF and MBF, whose activity is controlled by the binding of the repressor Whi5. Phosphorylation and removal of Whi5 by the cyclin-dependent kinase (CDK) Cln3-Cdc28 alleviates the Whi5-dependent repression on SBF and MBF, initiating entry into a new cell cycle. This Whi5-SBF/MBF transcriptional circuit is analogous to the regulatory pathway in mammalian cells that features the E2F family of G1 transcription factors and the retinoblastoma tumor suppressor protein (Rb). Here we describe genetic and biochemical evidence for the involvement of another CDK, Pcl-Pho85, in regulating G1 transcription, via phosphorylation and inhibition of Whi5. We show that a strain deleted for both *PHO85* and *CLN3* has a slow growth phenotype, a G1 delay, and is severely compromised for SBF-dependent reporter gene expression, yet all of these defects are alleviated by deletion of *WHI5*. Our biochemical and genetic tests suggest Whi5 mediates repression in part through interaction with two histone deacetylases (HDACs), Hos3 and Rpd3. In a manner analogous to cyclin D/CDK4/6, which phosphorylates Rb in mammalian cells disrupting its association with HDACs, phosphorylation by the early G1 CDKs Cln3-Cdc28 and Pcl9-Pho85 inhibits association of Whi5 with the HDACs. Contributions from multiple CDKs may provide the precision and accuracy necessary to activate G1 transcription when both internal and external cues are optimal.

## Abstract 2

Initiation of the cell division cycle in yeast is controlled by two distinct kinases that coordinately regulate the interaction of Whi5, a repressor of initiation, with histone deacetylases.

## Author Summary

Eukaryotic cells grow and divide by progressing through carefully orchestrated stages of the cell cycle characterized by stage-specific patterns of gene expression, DNA replication, and scission. How stage-specific gene expression is coordinated with cell cycle progression is only partially understood. The phase known as G1 marks the initiation of the cell cycle (called START in yeast) and involves the coordinated expression of more than 200 genes regulated by two transcription factors, SBF and MBF. The activity of SBF and MBF is restrained by binding of the repressor protein Whi5 to the two transcription factors early in G1 phase. Phosphorylation of Whi5 by G1-specific forms of the cyclin-dependent kinase (CDK) Cdc28 promotes dissociation of Whi5 from SBF and its export from the nucleus; this, in turn, releases SBF to activate G1-specific transcription. This G1 transcriptional circuit is analogous to that defined in mammals by the E2F family of transcription factors and the retinoblastoma (Rb) tumor suppressor protein. Rb further contributes to the repression of G1-specific transcription in mammals by recruiting histone deacetylases (HDACs), which are chromatin remodeling complexes that regulate promoter accessibility. Here, we show that regulation of G1-specific transcription in yeast also involves repressor-mediated recruitment of HDACs. We demonstrate that repression by Whi5 is modulated by both Cln-Cdc28 and a second G1-specific CDK, Pcl-Pho85, and further show that both kinases regulate the interaction of Whi5 with HDACs. We propose that regulation of the repressor by more than one G1-specific CDK ensures definitive inactivation of Whi5, a critical event for appropriate cell cycle initiation.

## Introduction

Cyclin-dependent protein kinases (CDKs) act as molecular machines that drive cell division, and cell cycle progression is dependent on oscillation between CDK active and inactive states. In *S. cerevisiae*, the CDK Cdc28 associates with nine different cyclin subunits to promote and coordinate a complex network of events necessary for smooth cell cycle transitions [1]. Irreversible commitment to a new round of cell division occurs toward the end of G1 phase in a process called Start in yeast. The analogous regulatory event is called the restriction point in mammalian cells [2],[3]. In yeast, three G1 cyclins, Cln1, Cln2, and Cln3, associate with Cdc28 to initiate events required for progression through Start. Passage through Start catalyzes a defined molecular program that initiates DNA replication, budding, spindle maturation, and chromosome segregation [3].

One key feature of Start in yeast, and G1 progression in other eukaryotic cells, is the induction of a transcriptional program involving over 200 genes, including those encoding the G1 (*CLN1*, *CLN2*, *PCL1*, and *PCL2*) and B-type cyclins (*CLB5* and *CLB6*) [4],[5]. G1/S phase-specific transcription depends on two heterodimeric transcription factors called SBF (Swi4,6 cell cycle box binding factor) and MBF (MluI binding factor). These complexes share a common regulatory subunit, Swi6, which is tethered to DNA via its binding partners, encoded by *SWI4* in SBF and *MBP1* in MBF [5]. At the well-studied *HO* locus, binding of the zinc-finger transcription factor Swi5 is followed by recruitment of the Swi/Snf chromatin remodeling complex and the SAGA histone acetyltransferase complex [6]–[8]. These events set the stage for SBF binding and recruitment of the SRB/mediator complex [6]. Importantly, subsequent recruitment of PolII and transcription initiation is dependent on CDK activity [9]. Although any one of the three G1 cyclins is sufficient to drive Start, genetic studies indicate a key role for Cln3-Cdc28 in activating SBF and MBF. At the same time Cln1 and Cln2 are required for the proper execution of other Start-related events such as budding and DNA synthesis. Cells lacking *CLN3* are large and severely delayed for onset of G1/S transcription, while ectopic induction of *CLN3* in small G1 cells activates transcription and accelerates passage through Start [10].

Start does not occur until cells have passed a critical cell size threshold, a barrier modulated by nutrient conditions, among other regulatory inputs [11]. A systematic analysis of cell size profiles for the entire set of yeast deletion mutants uncovered many new regulators of Start including Whi5 and implicated it as an inhibitor of G1/S-specific transcription [12],[13]. Whi5 occupies specific promoters early in G1 phase when CDK activity is low. However, Cdc28-dependent phosphorylation of both Whi5 and SBF/MBF late in G1 phase results in disengagement from SBF and nuclear export of Whi5 consequently leading to activation of SBF- and MBF-dependent transcription [12],[13].

Whi5 is proposed to function in a manner analogous to the well-characterized Rb family proteins in metazoans. E2F, the functional analog of SBF/MBF, regulates G1-specific gene expression required for passage through the restriction point [14]. E2F activity is restricted to late G1 phase because of inhibition by the retinoblastoma protein (Rb). Rb associates with E2F to restrain its activity until late G1, at which point stepwise phosphorylation of Rb by two CDKs, cyclin D-Cdk4/6 and cyclin E-Cdk2, causes the dissociation of Rb from E2F [15]. This process appears to be regulated by a positive feedback loop in which Rb phosphorylation by cyclinE-Cdk2 leads to further dissociation of Rb from promoters and enhancement of G1-transcription. At the molecular level, Rb interacts with both E2F and chromatin remodeling complexes such as histone deacetylases (HDACs) [16]–[18]. Rb appears to repress transcription through at least three distinct mechanisms: (1) Rb can bind directly to the activation domain of E2F thereby blocking its activity [19]; (2) recruitment of Rb can block the assembly of the pre-initiation complex thus inhibiting the activity of adjacent transcription factors [20] and; (3) Rb can recruit remodelers such as HDAC1 and BRG1 to modify chromatin structure. BRG1 is one of the human Swi/Snf adenosine triphosphatases (ATPases) that remodel nucleosomes by utilizing ATP to weaken the interactions between DNA and histones [16],[17]. The specific roles of different CDKs in regulating E2F-Rb function, however, remain unclear.

Another yeast CDK Pho85 was originally discovered as a regulator of phosphate metabolism, but has since been shown to play numerous roles in the regulation of cell division and other processes [21]–[23]. Ten genes encoding Pho85 cyclins (Pcls) have been identified and they appear to dictate substrate and functional specificity of Pho85 [24]–[26]. Expression of three Pcls, *PCL1*, *PCL2*, and *PCL9*, is restricted to G1 phase of the cell cycle [25]. Specifically, *PCL9* expression peaks early in G1, whereas maximal expression of *PCL1* and *PCL2* is observed at Start and is dependent largely on SBF [27]–[29]. Although Pho85 is not essential for viability, it is required for cell cycle progression in the absence of the Cdc28 cyclins *CLN1* and *CLN2* [29], and its absence leads to catastrophic morphogenic changes that culminate in a G2 arrest [30]. Consistent with this observation, inactivation of both Cdc28 and Pho85 CDKs specifically inhibits expression of G1-regulated genes involved in polarized growth [31].

As noted above, transcriptional repression by Rb has been linked to its interaction with histone modification complexes, in particular HDACs. Recent work highlights the importance of post-translational modifications of histone and nucleosome positioning in regulating gene expression [32],[33]. Histone acetylation neutralizes the positive charge generated by lysine-rich regions present in the N-terminal tails of histones, thereby disrupting nucleosome structure and increasing promoter accessibility [34]. As a result, many transcription activators have been shown to interact with histone acetyltransferases, whereas transcriptional repressors often associate with HDACs to promote nucleosome formation to occlude transcription factor binding [35],[36]. Histone deacetylation in *S. cerevisae* is mediated by a family of HDACs including Rpd3, Hda1, Hda2, Hos1, Hos2, and Hos3 [37]. Similar to their mammalian counterparts, some yeast HDACs are recruited to promoters by sequence-specific regulatory factors to repress gene expression. For example, the Rpd3 deacetylase complex is recruited to the *INO1* promoter by the DNA binding protein Ume6 [35],[38]–[40]. This recruitment results in local histone deacetylation and repression of *INO1* gene expression [41]. Hda1 is another example of this type of regulation, and is recruited to its target promoters by the repressor Tup1 [42].

In this study, we provide detailed mechanistic insights into Whi5-dependent regulation of G1-specific transcription and cell cycle progression. Specifically, we identify Whi5, to our knowledge, as the first demonstrated physiological substrate for the G1-specific Pcl9-Pho85 CDK and provide genetic and biochemical evidence supporting a direct role for Pho85 at Start. Furthermore, we show that in a manner similar to Rb in mammalian cells, Whi5-mediated repression involves the HDACs Rpd3 and Hos3. Dual phosphorylation of Whi5 by Cdc28 and Pho85 inhibits Whi5 activity in at least two ways. Both kinases appear to regulate interaction of Whi5 with different HDACs, whereas Cdc28 is also involved in disrupting Whi5 association with SBF and promoting its nuclear export [12],[13]. G1-specific CDKs thus are specialized to regulate different aspects of the same critical cell cycle event—inhibition of Whi5—resulting in definitive inactivation of the Whi5 repressor.

## Results

### A Synthetic Dosage Lethality Screen Identifies Whi5 as a Putative Substrate for the CDK Pho85

**Figure 1:**
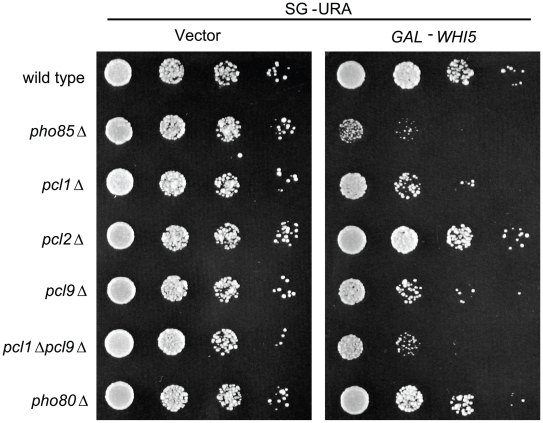
*WHI5* overexpression is toxic to strains compromised for Pho85 CDK activity. Isogenic wt (BY263), *pho85*Δ (BY391), *pcl1*Δ (BY628), *pcl2*Δ (BY451), *pcl9*Δ (BY694), *pcl1*Δ *pcl9*Δ (BY760), and *pho80*Δ (BY490) strains bearing either *GAL1-WHI5* (pBA1973) or empty vector control (pEG-H) were spotted in serial 10-fold dilutions on galactose media and incubated for 72 h at 30°C.

Synthetic dosage lethality (SDL) is a genetic assay that is based on the rationale that increasing levels of a protein may have no effect on the growth of an otherwise wild-type (wt) strain but may cause a measurable phenotype—such as lethality—in a mutant strain with reduced activity of an interacting protein [43],[44]. Previous studies suggest that SDL can be used effectively to identify novel enzyme targets and a genome-wide SDL screen in cells lacking Pho85 identified known targets of the CDK [23]. In addition to known substrates, several putative Pho85 targets were also identified, including the G1-specific transcription repressor Whi5 [24]. To further explore the role of Pho85 in G1 phase-specific transcription we examined the *WHI5-PHO85* SDL interaction in greater detail. As noted previously, Pho85 activity and substrate specificity depends on its interaction with cyclin subunits known as Pcls [25]. To implicate specific Pcl-Pho85 complexes in modulating Whi5 function we examined the effects of *WHI5* overexpression in cells lacking different Pcls (Figure 1). Similar to effects observed in *cln3*Δ and *cln1*Δ *cln2*Δ mutants [13], overexpression of *WHI5* resulted in growth inhibition of *pcl1*Δ and *pcl9*Δ deletion strains and this growth defect was exacerbated in a *pcl1*Δ *pcl9*Δ double mutant (Figure 1). Unlike *pcl1*Δ or *pcl*9Δ mutants, strains lacking *PCL2* or *PHO80* cyclins were not adversely affected by increased *WHI5* dosage suggesting that the *WHI5*-*PHO85* genetic interaction is dependent on the *PCL1,2* cyclin subfamily and more specifically on *PCL1* and *PCL9* (Figure 1). This observation is consistent with the fact that Pcl1 and Pcl9 (but not Pcl2) are the two G1-specific cyclins that localize to the nucleus [30],[45]. The growth phenotype seen in the plating assay was confirmed by measuring growth rates in liquid culture (unpublished data). On the basis of these results, Pcl1/9-Pho85 may contribute to Whi5 regulation in a manner similar to Cln3-Cdc28.

### Whi5 Is a Substrate for Pcl9-Pho85 Phosphorylation

**Figure 2:**
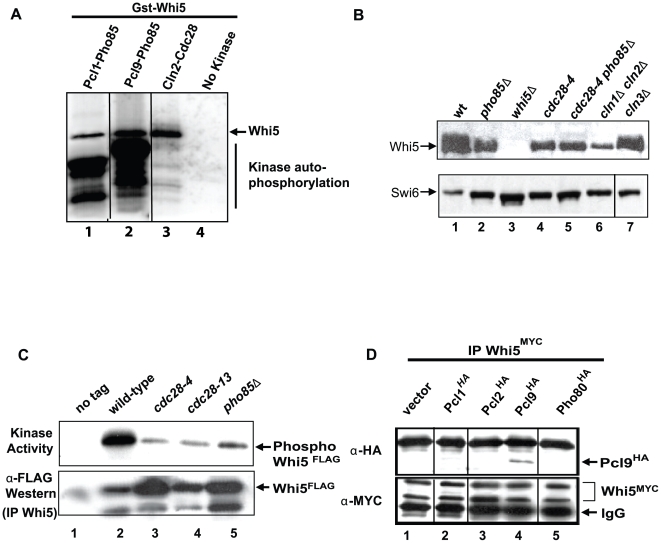
Whi5 is a substrate for Pcl9-Pho85 CDK-dependent phosphorylation. (A) In vitro phosphorylation of Whi5 by Pho85 kinase. Purified Whi5^GST^ and γ-^32^P-ATP were incubated alone (lane 4) or in the presence of recombinant Pcl1-Pho85 (lane 1), Pcl9-Pho85 (lane 2), or Cln2-Cdc28 (lane 3) kinases. Phosphorylated Whi5 protein was resolved by SDS-PAGE and autoradiography. (B) Slower-migrating forms of Whi5 are dependent on Cdc28 and Pho85. Cell extracts were prepared from wt (BY2507, lane 1), *pho85*Δ (BY4152, lane 2), *cdc28-4* (BY4153, lane 4), *cdc28-4 pho85*Δ (BY4154, lane 5), *cln1*Δ *cln2*Δ (BY4289, lane 6), and *cln3*Δ (BY4288, lane 7) strains expressing *WHI5^MYC^* along with a *whi5*Δ control strain (BY4454, lane 3). Cells were grown at 30°C (semipermissive temperature for *cdc28-4* strains) to log phase (Optical Density [OD] = 0.6) before harvesting. *cdc28-4* cells were placed at 37°C for 2 h to inactivate Cdc28 before harvesting. Whi5^MYC^ mobility was assessed by immunoblotting. (C) Whi5 associates with Pho85-dependent kinase activity. Wt (BY263) (lane 2), *cdc28-4* (BY465) (lane 3), *cdc28*-*13* (BY462) (lane 4), or *pho85*Δ (BY867) (lane 5) strains bearing a *GAL-WHI5^FLAG^* plasmid (pMT3586) or control vector control (pMT3164) were grown at 30°C (semipermissive temperature for *cdc28-4* and *cdc28-13* strains) in galactose media for 3 h. Whi5 complexes were recovered on anti-FLAG resin, incubated in kinase buffer with γ-^32^P-ATP at 30°C and resolved by SDS-PAGE. Capture of Whi5 protein was detected with anti-FLAG antibody. (D) Whi5 interacts with the Pcl, Pcl9. Anti-MYC immune precipitates of *WHI5^MYC^* strain lysates (BY2507) bearing either *PCL1^HA^* (pBA1820, lane 2), *PCL2^HA^* (pBA1821, lane 3), *PCL9^HA^* (pBA1822, lane 4), *PHO80^HA^* (pBA1823, lane 5), or a vector control (pBA330v, lane 1) were probed with 9E10 anti-MYC and 12CA5 anti-HA antibodies. The black lines in (A, B, and D) indicate empty lanes that were removed from the original blot.

The genetic interactions described above suggest Whi5 may be a direct target of Pho85. Evidence supporting this hypothesis is provided by protein microarray assays where Whi5 is phosphorylated in vitro by Pcl1-Pho85 [46]. We characterized the Whi5-Pho85 interaction biochemically by performing in vitro kinase assays using recombinant Pcl-Pho85 CDK complexes and purified Whi5 as substrate (Figure 2A). Incorporation of [^32^P] into Whi5 was not detected in the absence of CDKs (Figure 2A, lane 4). However, Whi5 phosphorylation was observed in the presence of Pcl1- and Pcl9-Pho85 (Figure 2A, lanes 1,2) and when compared to Cln2-Cdc28 kinase activity, Pho85 and Cdc28 phosphorylated Whi5 at similar levels in vitro (Figure 2A, lanes 1–3).

Previous studies revealed multiple Whi5 slow-migrating isoforms that correlate with its phosphorylation state [12],[47]. We examined the effect of various cyclin or CDK mutants on Whi5 mobility (Figure 2B). Because of genetic redundancy of Pcl cyclins [27], we were unable to reproducibly detect changes in Whi5 phosphoforms in cyclin mutant strains. Therefore, a Pho85 mutant was used to asses the phosphorylation status of Whi5. Consistent with previous findings [12],[13], slow migrating Whi5 isoforms present in asynchronous wt extracts (Figure 2B, lane 1) were modestly reduced in cells lacking *CLN3* (Figure 2B, lane 7) and completely absent in a *cln1*Δ *cln2*Δ double mutant (Figure 2B, lane 6), confirming that Whi5 phosphorylation depends on Cln-Cdc28 kinase complexes. Consistent with our SDL results and in vitro kinase assays, we observed a significant reduction in Whi5 mobility in extracts from a *pho85* mutant strain (Figure 2B, lane 2). Thus, similar to Cdc28, phosphorylation of Whi5 also depends on Pho85 in vivo.

To determine if Whi5 physically associates with Pho85 in yeast, we first assayed Whi5^FLAG^ immune complexes for kinase activity. A robust autophosphorylation activity was recovered from Whi5^FLAG^ immunoprecipitates derived from wt cell extracts when radiolabeled ATP was added to the immunoprecipitated sample (Figure 2C, lane 2). This activity was partially dependent on both *CDC28* and *PHO85* (Figure 2C, lanes 3–5). We also confirmed a physical interaction between Whi5 and Pcls using a co-immunoprecipitation assay (Figure 2D). Immunoprecipitation of Whi5^MYC^ from epitope-tagged cyclin extracts revealed a specific association between Pcl9 and Whi5 (Figure 2D, lane 4). We failed to reproducibly detect a physical interaction between Whi5 and Pcl1 (Figure 2D, lane 2) suggesting that Pcl9-Pho85 is the primary Whi5 CDK. Taken together, the phosphorylation and co-immunoprecipitation assays strongly suggest that, in addition to Cdc28, Pho85 also phosphorylates Whi5. Furthermore these results identify Whi5 as the first reported substrate for Pcl9-Pho85, one of two Pcls whose activity is restricted to early G1 phase.

**Figure 3:**
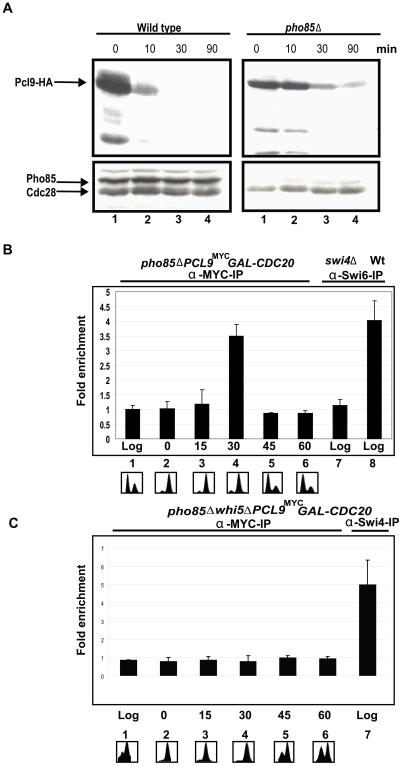
Pcl9 localizes to G1-specific promoters in a cell cycle-dependent manner. (A) Pho85 regulates Pcl9 protein stability. Wt (BY263) and *pho85*Δ strains (BY391) harboring a *GAL1-PCL9^HA^* plasmid (pBA2112) were grown to exponential phase in galactose media (lane 1). *PCL9* expression was repressed by addition of glucose to final concentration of 2% and cells were harvested 10 (lane 2), 30 (lane 3), and 90 (lane 4) min after addition of glucose. Pcl9 abundance was assessed by immunoblotting using 12CA5 anti-HA antibodies. (B) Pcl9 localizes to SBF-dependent promoters. An exponentially growing *GAL1-CDC20 pho85*Δ*PCL9^MYC^*strain (BY4148, lane 1) was arrested at M/G1 phase in glucose-containing medium (lane 2). Cultures were harvested 15 (lane 3), 30 (lane 4), 45 (lane 5), and 60 (lane 6) min after release from *CDC20*-induced arrest in galactose medium. Cell cycle progression was monitored by FACS analysis. Anti-MYC and anti-Swi6 ChIPs from the indicated strains were analyzed for *CLN2* promoter sequences by quantitative RT-PCR. (C) In a strain lacking Whi5, *GAL1-CDC20 pho85*Δ *whi5*Δ*PCL9^MYC^*, Pcl9 no longer localizes to the *CLN2* promoter. Anti-Swi4 ChIPs are shown as a positive control.

Whi5 associates indirectly with G1 phase-regulated promoters through interaction with SBF and MBF. Interactions with these transcription factors and subsequent promoter binding are disrupted by CDK-dependent phosphorylation [12],[13]. Because Whi5 appears to be a Pho85 substrate, we assessed the occupancy of SBF promoters by Pcl9. To date, cyclins have not been detected at yeast promoters. Pcl9 is normally an unstable short-lived protein [27]; however, similar to other cyclins, Pcl9 turnover appears to be catalyzed in part by its cognate CDK, Pho85 (Figure 3A) [48]. Therefore, to test Pcl9 promoter localization in a more sensitive genetic background, we performed ChIP (Chromatin immunoprecipitation) experiments in a *pho85*Δ strain (Figure 3B). The highest levels of *CLN2* promoter DNA were detected in Pcl9^MYC^ immune complexes 30 min following release from a metaphase-anaphase arrest (Figure 3B). The Pcl9-chromatin association was no longer detectable 45 min after *GAL-CDC20* induction indicating that the interaction is short-lived and transient as predicted for a regulator of Start. The association was Whi5-dependent since Pcl9 was not detected at the *CLN2* promoter in a strain lacking Whi5 (Figure 3C). The localization of Pcl9 to *CLN2*, a G1 promoter, is consistent with a direct role for Pcl9-Pho85 in regulating G1 transcription.

### Pcl9-Pho85 Regulates Whi5 Function via Phosphorylation

**Figure 4:**
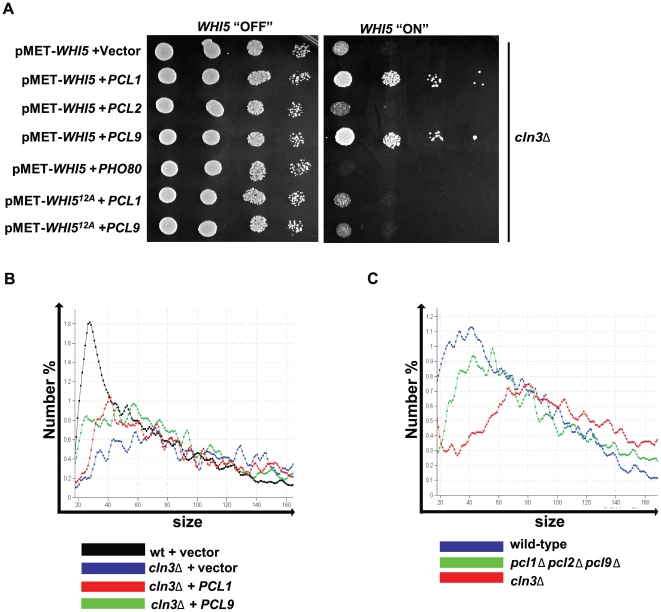
*PHO85* affects growth and cell size defects associated with *cln3*Δ. (A) Ectopic *PCL1* and *PCL9* expression alleviates *WHI5* toxicity in a *cln3*Δ strain. A *cln3*Δ strain (BY653) bearing a methionine-repressible *WHI5^GST^* (pBA1975) or *WHI5^12A-GST^* low-copy plasmid (pBA2249) along with an additional vector control (pBA330v), *PCL1^HA^* (pBA1820), *PCL2^HA^* (pBA1821, *PCL9^HA^*, pBA1822), or *PHO80^HA^* (pBA1823) construct were spotted in serial 10-fold dilutions on media supplemented with or lacking methionine (*WHI5* “OFF”, *WHI5* “ON,” respectively) and incubated for 72 h at 30°C. (B) *PCL1* and *PCL9* cyclins modulate cell size. Cell size distributions were analyzed for wt (BY263) and *cln3*Δ strains (BY653) bearing vector control (pBA330v), *PCL9^HA^* (pBA1822), or *PCL1^HA^* (pBA1820) plasmids. The median cell volume based on three replicates was: 42.33 fl±1.13 (wt+vector control); 71.78 fl±1.43 (*cln3*Δ+vector control); 55.67 fl±1.66 (*cln3*Δ+*PCL1*); 54.25 fl±1.21 (*cln3*Δ+*PCL9*). (C) Cells lacking *PHO85* G1 cyclins exhibit an enlarged cell size. Cell size distributions were analyzed for wt (BY263), *pcl1*Δ *pcl2*Δ *pcl9*Δ (BY764), and *cln3*Δ strains (BY653). The median cell volume based on three replicates was: 46.73 fl±0.63 (wt); 53.96 fl±0.75 (*pcl1*Δ *pcl2*Δ *pcl9*Δ); 72.72 fl±1.22 (*cln3*Δ).

As mentioned above, *cln3*Δ mutants arrest in G1 phase as large unbudded cells in response to increased *WHI5* dosage, indicating that Whi5 is a dose-dependent regulator of Start. Therefore, if Pho85 and Cdc28 function analogously to inhibit Whi5 activity, we predict that elevated Pho85 kinase activity would antagonize the toxic effects of *WHI5* overexpression and suppress the growth defects observed in a *cln3*Δ mutant. To test this prediction, high copy plasmids expressing *PCL1*, *PCL2*, *PCL9*, or *PHO80* were introduced into a *cln3*Δ strain expressing *WHI5* from a conditional *MET25* promoter (Figure 4A). Plasmid-based expression of Pcls and Whi5 was confirmed by immunoblotting (Figure S1). Induction of *WHI5* expression in a *cln3*Δ mutant resulted in cell death whereas overexpression of *PCL1* or *PCL9* partially suppressed this toxicity and restored growth (Figure 4A). Consistent with results from SDL analyses (Figure 1), this suppression was specific to *PCL1* and *PCL9* since neither *PCL2* nor *PHO80* were able to function effectively in the assay (Figure 4A). Furthermore, *PCL1/9*-mediated suppression was dependent on phosphorylation since growth of a *cln3*Δ mutant expressing a nonphosphorylatable form of *WHI5* (Whi512A) [13] could not be restored (Figure 4A). These genetic results corroborate the biochemical evidence that Pcl-Pho85 regulates Whi5 activity through phosphorylation.

Given its effect on *WHI5* overexpression, we next examined *PCL* effects on other *CLN3*-associated phenotypes. *CLN3* is required to activate G1-specific transcription once cells have achieved a critical size [49]–[51]. A *cln3*Δ mutant exhibits a large cell size phenotype because of its inability to inhibit Whi5 and activate Start-specific transcription [12],[13]. Ectopic expression of *PCL1* or *PCL9* reduced *cln3*Δ cell size to an intermediate level between that of wt and *cln3*Δ cells (Figure 4B). Conversely, deletion of *PCL9*, *PCL1*, and the partially redundant cyclin *PCL2* resulted in a cell size increase (Figure 4C). These results suggest that Pcl-Pho85 and Cln3-Cdc28 share a common role in cell cycle progression to regulate Whi5 activity and promote passage through Start.

### *CDC28* and *PHO85* Function in Parallel Pathways to Regulate Whi5 Function

**Figure 5:**
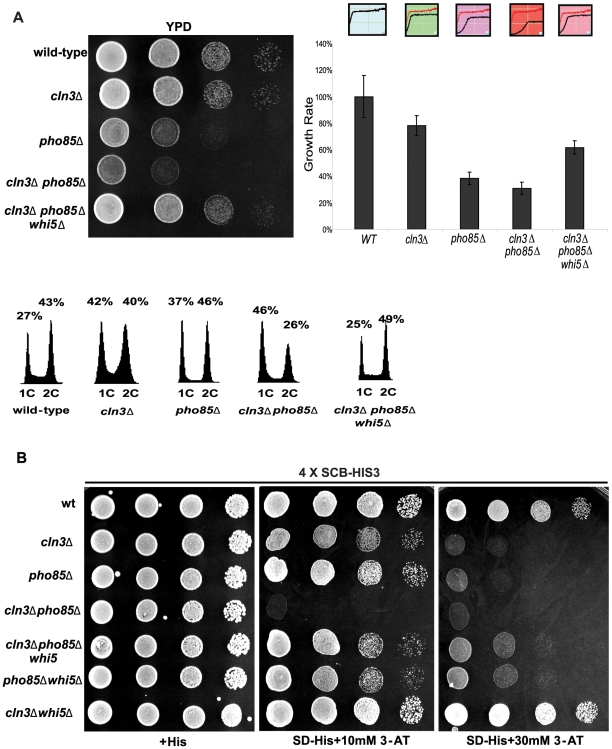
*PHO85* regulates G1 transcription via *WHI5*. (A) The G1 delay phenotype associated with a *cln3*Δ *pho85*Δ strain is dependent on *WHI5*. Wt (BY263), *cln3*Δ (BY653), *pho85*Δ (BY391), *cln3*Δ *pho85*Δ (BY4291), and *cln3*Δ *pho85*Δ *whi5*Δ (BY4292) strains were spotted in serial 10-fold dilutions on rich media (YPED) and incubated for 24 h at 30°C. DNA content of exponentially growing cultures was determined by FACS analysis. Liquid growth assays were also performed for these strains and growth rate is reported relative to wt as shown in the bar graph. Graphical representations of growth rates are shown above the bar graph as line plots, where the upper red line represents the growth of WT and the black line shows the growth of each mutant. (B) A *cln3*Δ *pho85*Δ strain exhibits defects in SCB-driven gene expression. Wt (BY4302), *cln3*Δ (BY4303), *pho85*Δ (BY4304), *cln3*Δ *pho85*Δ (BY4305), *cln3*Δ *pho85*Δ *whi5*Δ (BY4306), *pho85*Δ*whi5*Δ (BY4308), and *cln3*Δ*whi5*Δ (BY4307) strains harboring an integrated *SCB*-*HIS3* reporter were spotted in serial 10-fold dilutions on histidine-containing medium or media lacking histidine and supplemented with 10 or 30 mM 3-AT. Plates were incubated at 30°C for 48 h. We note that the synthetic growth defect of a *cln3 pho85* mutant is most pronounced on rich medium (A), and is not as evident when strains are grown on minimal medium.

To determine if Pcl-Pho85 and Cln3-Cdc28 might function in parallel to regulate Start, we first tried to test whether *pcl9*Δ *cln3*Δ or *pcl1*Δ *pcl9*Δ *cln3*Δ strains showed any synthetic growth defects. As expected, no growth defects were observed, probably because of the redundant effects of other Pcls [27]. Unlike the Cdc28 cyclins, which shows distinct cell cycle expression patterns, most Pcls are expressed at all cell cycle stages [25]. We then examined the phenotype of a *pho85*Δ *cln3*Δ double mutant. Cells lacking *cln3*Δ are larger than wt cells but do not display overt defects in growth rate while *pho85*Δ mutants are slow growing (Figure 5A). However, *pho85*Δ*cln3*Δ double mutants exhibited a more pronounced growth defect compared to single mutants and analysis of DNA content revealed that the *pho85*Δ *cln3*Δ double mutant cells accumulated in G1 phase with predominantly unreplicated DNA (Figure 5A). Importantly, deleting *WHI5* overcame both the cell cycle progression and growth defects observed in the absence of both *CLN3* and *PHO85*. Notably, a *pho85*Δ *cln3*Δ *whi5*Δ triple mutant exhibited a growth rate similar to a *cln3*Δ single mutant indicating that Pcl-Pho85 and Cln3-Cdc28 function in separate yet converging pathways to regulate Whi5 function and, by extension, G1 cell cycle progression (Figure 5A). These observations also hold true under liquid growth conditions as shown. *WHI5*-dependent suppression appears to be specific to the *pho85*Δ *cln3*Δ phenotype because *WHI5* deletion was unable to rescue 53 additional synthetic lethal interactions involving *PHO85* (Table S1; D.Q. Huang and B.J. Andrews, unpublished data).

Given that Whi5 represses SBF- and MBF-specific transcription, we asked whether *PHO85* affects SBF-driven reporter gene expression. A reporter gene consisting of tandem SCB consensus element repeats fused upstream of the *HIS3* coding region was constructed and integrated into wt, *cln3*Δ, and *pho85*Δ strains. Previous work has shown that this reporter provides a highly specific read-out for SBF-dependent transcription [13],[52]. Growth on medium lacking histidine supplemented with 3-aminotriazole (3-AT) was used to assess SBF transcriptional activity (Figure 5B). Even though cells lacking *PHO85* were moderately sensitive to higher concentration (5 mM) of 3-AT (unpublished data), both *cln3*Δ and *pho85*Δ mutants showed no growth in media containing 30 mM 3-AT indicating that SBF transcription is impaired in these mutants, whereas growth of wt cells was unaffected [13]. Furthermore, defects in SCB-driven gene expression were more pronounced in the *pho85*Δ *cln3*Δ double mutant (at 10 mM 3-AT, Figure 5B). Consistent with the genetic interactions described above (Figure 5A), SBF-dependent reporter activity was restored in *pho85*Δ *cln3*Δ mutants when *WHI5* was deleted (Figure 5B). However, *WHI5* deletion only partially rescued the growth defect in *pho85*Δ cells at 30 mM of 3-AT (Figure 5B). The Whi5-independent 3-AT sensitivity of *pho85*Δ cells may be due to unregulated Gcn4 in the absence of *PHO85*, since *GCN4* is induced by 3-AT and Pho85 has been shown to regulate Gcn4 stability [53],[54]. Nonetheless, these data suggest that, like Cln3-Cdc28, Pcl-Pho85 modulates SBF activity through Whi5.

**Figure 6:**
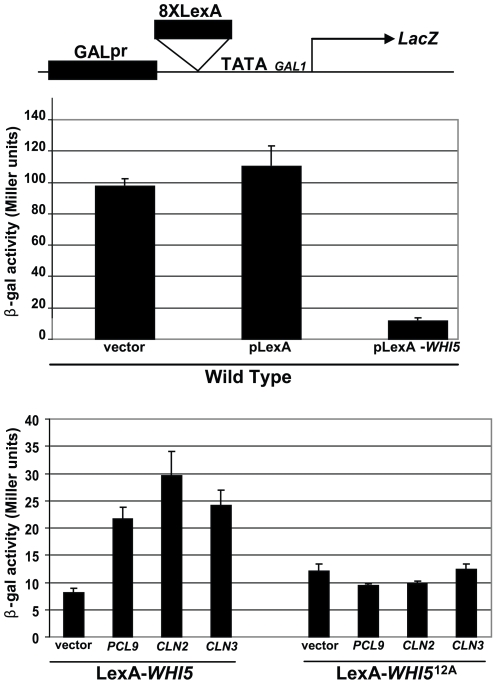
Whi5-mediated transcriptional repression is antagonized by *PHO85* and *CDC28*. A reporter gene consisting of eight LexA binding sites flanked by the *GAL1* promoter and the *LacZ* coding sequence was constructed (pBA1976). β-galactosidase activity (upper histogram) was measured in a wt strain (BY263) bearing the *LacZ* reporter along with one of the following: a vector control (pBA230v); a LexA expressing plasmid (pLexA; pBA1977); or a construct expressing a LexA-Whi5 fusion protein (pLexA-*WHI5*; pBA1978). β-galactosidase activities were also assayed (lower histogram) in a wt strain harboring the *LacZ* reporter construct alone (vector control; pBA1976) or overexpressing the G1 cyclins, *PCL9* (pBA1974), *CLN2* (pBA2247), or *CLN3* (pBA2248) in the presence of LexA-Whi5 (pBA1978) or LexA-Whi5^12A^ (pBA1979) fusion proteins.

We next interrogated the effects of CDK activity on Whi5-mediated transcriptional repression (Figure 6). A construct expressing a LexA DNA binding domain fused to *WHI5* was introduced into a strain harboring a *LacZ* reporter gene containing LexA binding sites in its promoter (Figure 6). Consistent with its role as a negative regulator of G1-specific transcription, a ∼10-fold reduction in β-galactosidase activity was observed in cells expressing the LexA-Whi5 fusion protein compared to a vector control (Figure 6). Overexpression of *PCL9*, *CLN3*, or *CLN2* restored *LacZ* expression to intermediate levels indicating that activation of either *CDC28* or *PHO85* was capable of antagonizing Whi5 function in this assay (Figure 6). Consistent with suppression of *WHI5*-mediated growth defects (Figure 4), inhibition of Whi5 activity was dependent on phosphorylation since *LacZ* expression could not be restored in cells harboring an unphosphorylatable LexA-Whi5^12A^ fusion protein (Figure 6).

### Pho85 Does Not Regulate Whi5 Localization or Its Interactions with G1-Specific Transcription Complexes

**Figure 7:**
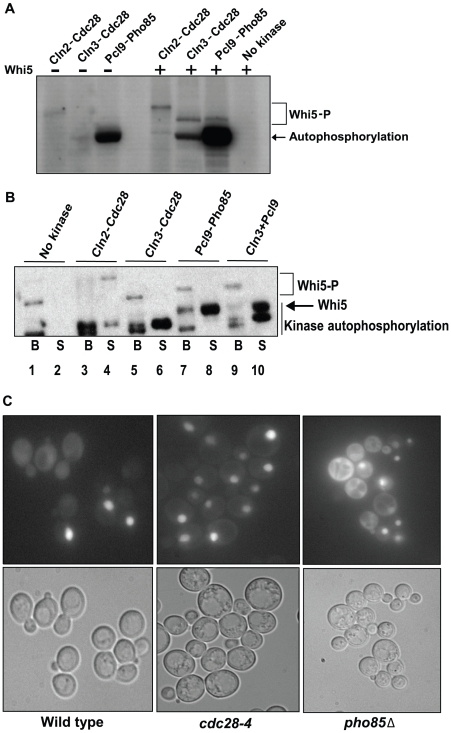
Pho85 does not affect known Whi5 regulatory mechanisms. (A) Determination of relative Cdc28 and Pho85 kinase activity. In vitro kinase assays using varying amounts of recombinant Cln2-Cdc28, Cln3-Cdc28, and Pcl9-Pho85 in the absence (lane 1–3) or presence of purified Whi5 (lanes 5–8) were conducted and the degree of Whi5 phosphorylation was determined by SDS-PAGE and autoradiography. Purified Whi5 and γ-^32^P-ATP were incubated in the absence of kinase in lane 8, and lane 4 is empty. A 3 µM final concentration of Cln3-Cdc28 and Pcl9-Pho85 and a 60 nM final concentration of Cln2-Cdc28 give similar amounts of ^32^P-incorporation in Whi5, although phosphorylation by Cln2-Cdc28 caused Whi5 to migrate more slowly than Whi5 phosphorylated by Cln3-Cdc28 or Pcl9-Pho85. The concentration of kinase used in (B) was based on these experiments. (B) Cln3-Cdc28 and Pcl9-Pho85 do not influence Whi5-SBF complex stability. A preassembled recombinant Whi5-Swi4^FLAG^-Swi6 complex bound to anti-FLAG resin was incubated with Cln2-Cdc28, Cln3-Cdc28, Pcl9-Pho85, or both Cln3-Cdc28 and Pcl9-Pho85 in the presence of radiolabeled ATP. After washing, proteins in the bound and supernatant fractions were identified by autoradiography. (C) Subcellular localization of Whi5 in *cdk* mutant strains. Wt (BY263), *pho85*Δ (BY391), and *cdc28-4* strains (BY465) expressing *WHI5^GFP^* from a methionine-repressible promoter (pBA1981) were examined for Whi5^GFP^ fluorescence. Representative fields are shown.

Cln2-Cdc28 activity was previously shown to disrupt recombinant Whi5-SBF complexes in vitro [13], but Cln3-Cdc28 and Pho85 kinases had not been assessed for this activity. A preassembled recombinant Whi5-Swi4^FLAG^-Swi6 complex bound to anti-FLAG resin was incubated with purified kinases in the presence of radiolabeled ATP and separated into soluble (Figure 7B, labeled “S”) and bound fractions (Figure 7B, labeled “B”). Equivalent amounts of kinase were approximated on the basis of in vitro kinase activity (Figure 7A, and Materials and Methods). As expected, Cln2-Cdc28 phosphorylation caused most of the SBF-bound Whi5 to be released into the soluble fraction (Figure 7B, lanes 3 and 4). In contrast, we failed to observe dissociation of Whi5 from SBF in the presence of Cln3- or Pcl9-CDK complexes (Figure 7B, lanes 5–10). In addition to negatively regulating the interaction of Whi5 with SBF, Cdc28 also controls its localization [13]. Unlike Cln-Cdc28 phosphorylation, which promotes Whi5 export from the nucleus, deletion of *PHO85* did not dramatically affect the subcellular localization of Whi5 (Figure 7C). Together, these results suggest that Pho85 must regulate Whi5 function through alternate mechanisms.

### Mechanism for Whi5-Mediated Transcriptional Repression by Pho85

**Figure 8:**
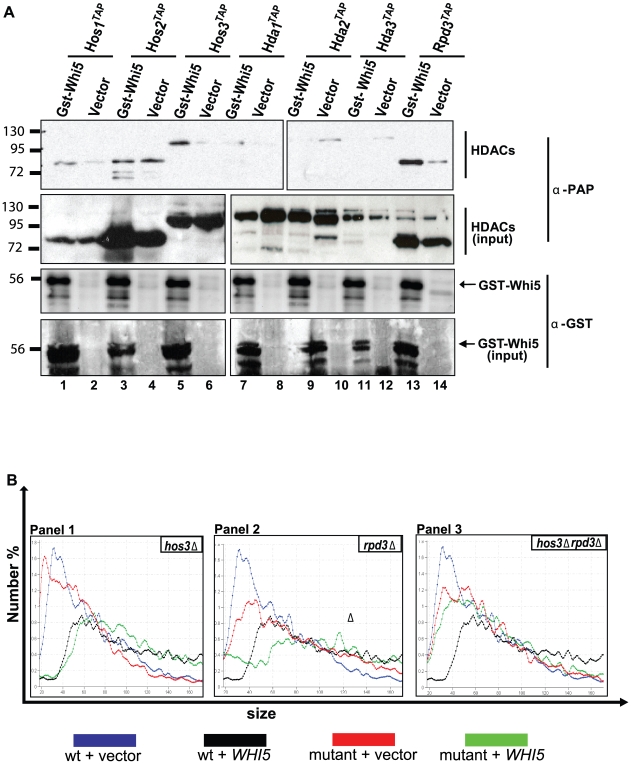
Whi5 function is dependent on HDAC activity. (A) Whi5 associates with Hos3 and Rpd3. Lysates prepared from the indicated epitope-tagged HDAC strains (BY4309–4315) harboring a vector control (pEG-H) or construct expressing *WHI5^GST^* (pBA1973) were incubated with glutathione sepharose beads. Whi5^GST^ -HDAC interactions were detected by immunoblot using α-GST and α-PAP antibodies. (B) Hos3 and Rpd3 modulate Whi5 cell size effects. A plasmid expressing *WHI5* (pBA1980) or vector control (pBA230v) were introduced into wt (BY263), *hos3*Δ (BY4293), *rpd3*Δ (BY4294), and *hos3*Δ *rpd3*Δ (BY4295) strains, and cell size distributions were measured. Each panel corresponds to a specific mutant and wt distributions are superimposed in each panel. The median cell volume based on three replicates was: 42.06 fl±1.09 (wt+vector control, blue); 73.12 fl±1.16 (wt+*WHI5*, black); 30.57 fl±1.23 (*hos3*Δ+vector control, panel 1, red); 71.35 fl±1.59 (*hos3*Δ+*WHI5*, panel 1, green); 51.20 fl±1.73 (*rpd3*Δ+vector control, panel 2, red); 69.75 fl±2.79 (*rpd3*Δ+*WHI5*, panel 2, green); 45.62 fl±1.22 (*hos3*Δ *rpd3*Δ+vector control; panel 3, red); 50.26 fl±1.14 (*hos3*Δ *rpd3*Δ+*WHI5*, panel 3, green).

We next explored what additional mechanism might explain Pcl- and Cln3-mediated regulation of Whi5 activity. Functional conservation clearly extends to Whi5 and its metazoan analogue Rb [14]. Since Rb represses transcription, in part, through recruitment of HDACs, we used a batch affinity chromatography assay to test for physical interactions between a Whi5^GST^ ligand and tandem affinity tagged HDACs (Figure 8A). Specific interactions between Whi5 and Hos3, Rpd3, and, to a lesser extent, Hos1 were identified (Figure 8A, lanes 1, 5, 13) suggesting that, like Rb, Whi5-dependent transcriptional repression involves recruitment of HDACs. This observation is consistent with previous work that detected Rpd3 at the *PCL1* promoter using a ChIP assay [55]. Furthermore, *HOS3* and *RPD3* were required for *WHI5* dose-dependent effects on cell size. Like wt cells, strains lacking either *HOS3* (Figure 8B, panel 1) or *RPD3* (Figure 8B, panel 2) also exhibited a dose-dependent increase in cell size in response to *WHI5* overexpression. However, additional cell size effects were not observed in strains lacking both HDACs, suggesting that Hos3 and Rpd3 regulate Whi5 function synergistically (Figure 8B, panel 3).

**Figure 9:**
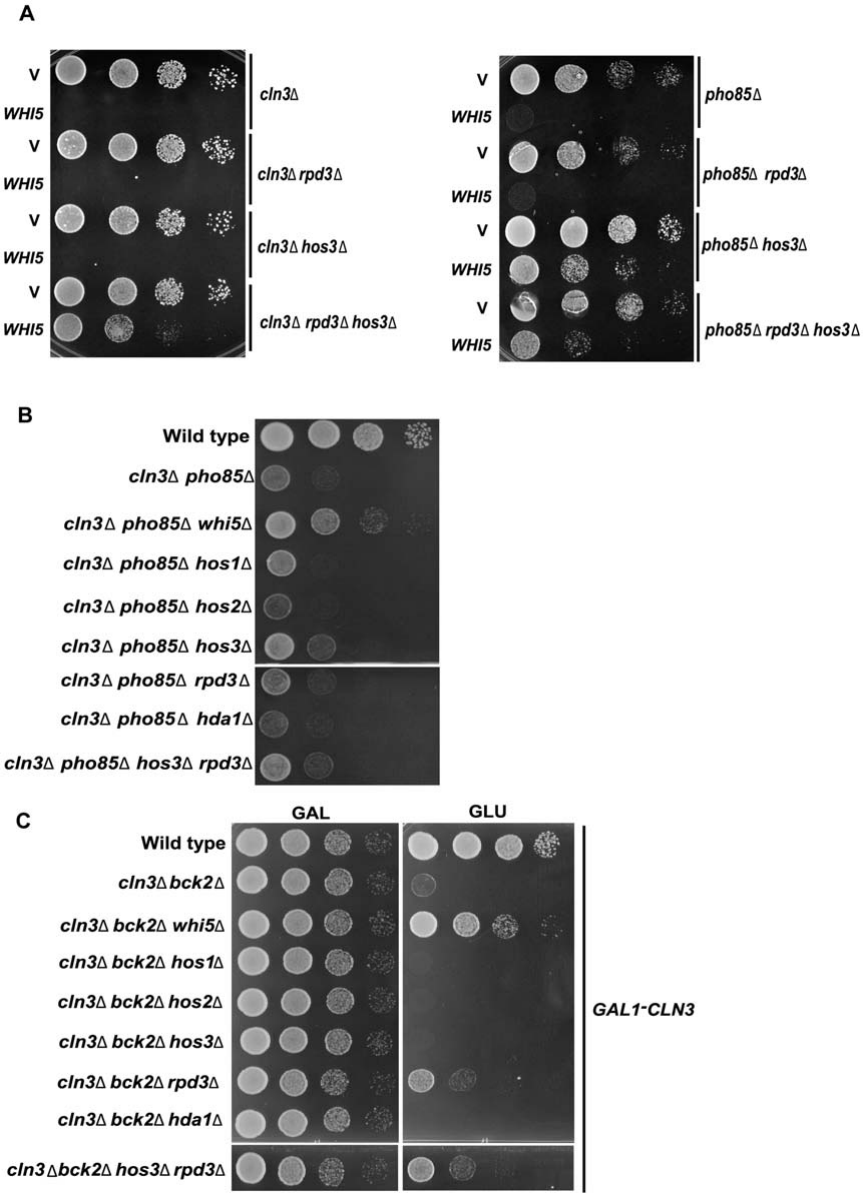
*WHI5* toxicity is dependent on *HOS3* and *RPD3*. (A) *cln3*Δ (BY4290), *cln3*Δ *rpd3*Δ (BY4297), *cln3*Δ *hos3*Δ (BY4296), and *cln3*Δ *rpd3*Δ *hos3*Δ (BY4298) strains harboring a methionine-repressible *WHI5* construct (pBA1975) or vector control (pBA228v) were spotted in serial 10-fold dilutions on medium lacking methionine. In a similar experiment, *pho85*Δ (BY391), *pho85*Δ *rpd3*Δ (BY4300), *pho85*Δ *hos3*Δ (BY4299), and *pho85*Δ *rpd3*Δ *hos3*Δ (BY4301) strains bearing a galactose-inducible *WHI5* plasmid (pBA1973) or appropriate vector control (pEG-H) were spotted in serial 10-fold dilutions on galactose-containing medium. Plates were incubated at 30°C for 48 h. (B) Deletion of *HOS3* partially restores growth of a *cln3*Δ *pho85*Δ strain. The indicated strains (BY263; BY4291, BY4292, BY4455–4461) were spotted in serial 10-fold dilutions on rich medium (YPED) and incubated at 30°C for 48 h. (C) Deletion of *RPD3* and *HOS3* partially restore viability of a *cln3*Δ *bck2*Δ strain. The indicated strains (BY4741; BY2948, BY4462–4468) were spotted in serial 10-fold dilutions on glucose-containing medium (YPED) to repress *CLN3* expression. Strains were also spotted on medium containing galactose as a control. Plates were incubated at 30°C for 72 h.

If HDACs are required for Whi5 function, then strains lacking HDAC function should be resistant to toxic effects associated with *WHI5* overexpression. Consistent with this prediction, the growth defect caused by *WHI5* overproduction in a *cln3*Δ was alleviated by the deletion of *HOS3* and *RPD3* (Figure 9A). Deletion of *HOS3* alone rescued *WHI5* toxicity in a *pho85*Δ strain while a *cln3*Δ mutant required deletion of both *HOS3* and *RPD3* in order to tolerate increased dosage of *WHI5* (Figure 9A).

Given that Whi5 appears to be acting through HDACs, we predicted that deletion of *HOS3* and *RPD3* should phenocopy those genetic interactions seen in *whi5*Δ mutants. We first tested various HDAC deletion strains for suppression of the slow growth phenotype of a *pho85*Δ*cln3*Δ mutant. As for *WHI5*, deletion of *HOS3* and *RPD3* partially suppressed the growth defect seen in the *pho85*Δ*cln3*Δ double mutant strain (Figure 9B). Suppression was specific to *HOS3* and *RPD3* because deletion of other HDACs showed no suppression, and the growth rate of the *pho85*Δ*cln3*Δ*hos3*Δ strain was not improved by subsequent deletion of *RPD3* and vice versa (Figure 9B).

**Figure 10:**
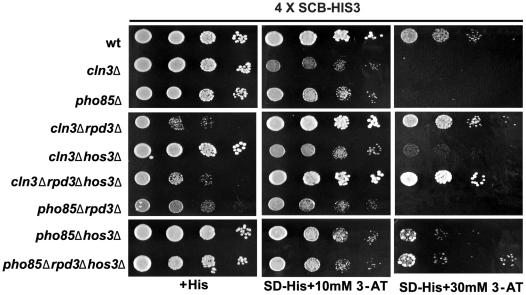
Repression of gene expression by Whi5 is dependent on *HOS3* and *RPD3*. The growth defects of *cln3*Δ and *pho85*Δ strains can be rescued by removing *RPD3* and *HOS3* in SCB-driven gene expression. Wt (BY4302), *cln3*Δ (BY4303), *pho85*Δ (BY4304), *cln3*Δ *rpd3*Δ (BY4297), *cln3*Δ *hos3*Δ (BY4296), *cln3*Δ*rpd3*Δ*hos3*Δ (BY4298), *pho85*Δ*rpd3*Δ (BY4300), *pho85*Δ*hos3*Δ (BY4299), and *pho85*Δ*rpd3*Δ*hos3*Δ (BY4301) strains harboring an integrated *SCB*-*HIS3* reporter were spotted in serial 10-fold dilutions on histidine-containing medium or media lacking histidine and supplemented with 10 or 30 mM 3-AT. Plates were incubated at 30°C for 48 h.

We next asked if deletion of HDACs might overcome the Start arrest seen in cells lacking both *CLN3* and *BCK2*, another regulator of G1 transcription that functions in parallel with *CLN3* [56]. A *cln3*Δ*bck2*Δ*whi5*Δ triple mutant grows as vigorously as wt, placing *WHI5* downstream of both upstream activators of G1 transcription [13]. Interestingly, deletion of *RPD3* partially restored growth in the *cln3*Δ*bck2*Δ strain providing further evidence for an HDAC requirement in Whi5-mediated transcriptional repression (Figure 9C). Neither subsequent deletion of *HOS3* nor deletion of other HDACs affected growth appreciably (Figure 9C). We also employed the *SCB-HIS3* assays used above to explore SBF-driven reporter gene expression in the HDAC mutants (Figure 10). As expected, deletion of *RPD3* rescued the growth defects of *cln3*Δ *SCB-HIS3* cells in the presence of both 10 mM and 30 mM of 3-AT, whereas *HOS3* gene knockout had a marginal but additive effect. In contrast, the growth of *pho85*Δ cells was slightly rescued by deletion of *HOS3* but not *RPD3* providing further evidence for Pho85 acting specifically through Hos3. Because of difficulties in detecting HDACs at promoters, we were unable to confirm these observations in vivo.

**Figure 11:**
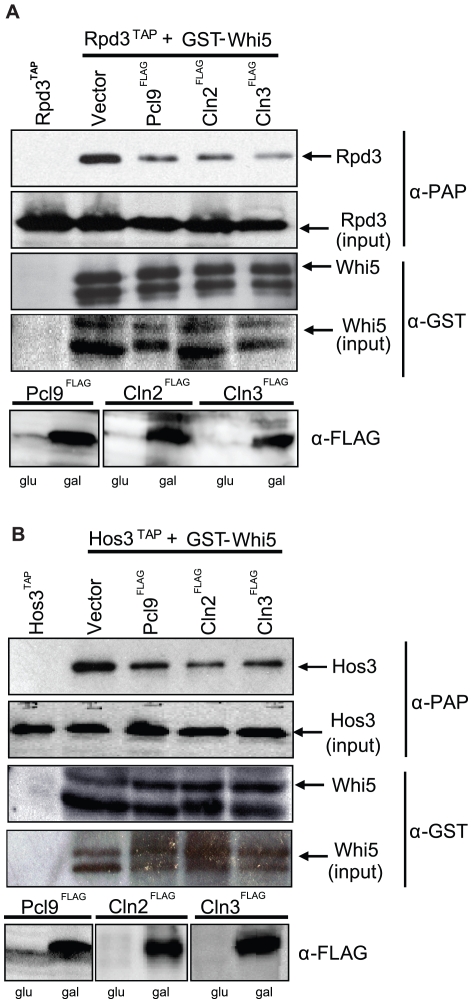
CDK activity antagonizes Whi5-HDAC interactions. (A) Pho85 and Cdc28 activity inhibits interaction between Whi5 and Rpd3. *PCL9^FLAG^* (pBA1974), *CLN2^FLAG^* (pBA2247), *CLN3^FLAG^* (pBA2248), or a vector control (pMT3164) were introduced into a strain harboring *RPD3^TAP^* at the chromosomal locus (BY4315) and a *WHI5^GST^* plasmid (pBA1973). Cyclin expression was confirmed by immunoblot using anti-FLAG antibodies. Lysates were incubated with glutathione sepharose beads. Whi5^GST^-Rpd3^TAP^ interactions were detected by immunoblot using α-GST and α-PAP antibodies, (B) Pho85 and Cdc28 activity inhibits interaction of Whi5 and Hos3. Experiments were conducted as described in (A) but using a strain bearing *HOS3^TAP^* at the chromosomal locus.

We also performed co-immunoprecipitation assays using affinity tagged *RPD3* and *HOS3* strains and observed an obvious decrease in Rpd3 and Hos3 in Whi5 precipitates from strains harboring increased levels of Pcl9, Cln2, or Cln3 cyclins (Figure 11A and 11B). Together, our genetic and biochemical results suggest that Pho85 may preferentially influence Whi5-Hos3 activity, whereas Cln3-Cdc28 is required for inhibition of both Rpd3 and Hos3.

## Discussion

Whi5 is a critical cell cycle regulator that links CDK activity in G1 phase to the broad transcriptional program that accompanies commitment to cell division. We provide substantial evidence that the multifunctional Pho85 CDK is an important regulator of Whi5 activity and G1 phase-specific transcription including: (1) Whi5 is phosphorylated and antagonized by Pho85 and is the first reported substrate for the G1-specific CDK complex, Pcl9-Pho85; (2) the activity of an SBF-dependent promoter is influenced by *PHO85*; (3) the Pcl9 cyclin binds to SBF-regulated promoters; (4) the repressor function of Whi5 is mediated through the HDACs Hos3 and Rpd3; and (5) HDAC-Whi5 association is regulated by G1-specific forms of both the Pho85 and Cdc28 CDKs. We therefore conclude that timely and efficient release from Whi5 inhibition and subsequent G1/S cell cycle progression requires the concerted activity of both Cdc28 and Pho85.

Several lines of evidence point to common roles for Pho85 and Cdc28. For example, a burst of both G1-specific Cdc28 and Pho85 activity is essential for cellular morphogenesis. A strain lacking the G1-specific cyclins, *CLN1*, *CLN2*, *PCL1*, and *PCL2*, undergoes a catastrophic morphogenic change and fails to establish polarized cell growth and cytokinesis [30]. Consistent with these observations, a chemical genomic analysis demonstrated that expression of genes involved in polarized cell growth was sensitive to simultaneous inhibition of both kinases, but not either single kinase [31]. A functional connection between Pho85 and Cdc28 is further supported by independent genetic and biochemical analyses that identify common targets phosphorylated by both kinases [45],[46],[48],[57]–[59].

Despite the clear functional overlap for G1-specific forms of Cdc28 and Pho85 in controlling morphogenesis, up to now, a direct role for Pho85 in cell cycle commitment and G1 phase-specific transcription has remained unclear. We discovered that, like Cdc28, Pho85 activates G1 transcription through inhibition of the Whi5 repressor. While the two kinases collaborate to control certain facets of Whi5 regulation, they are also specialized to modulate Whi5 function by distinct mechanisms. We have defined a novel HDAC-dependent mechanism that impinges on Whi5 function and implicates both Pho85 and Cdc28 as regulators of this process.

**Figure 12:**
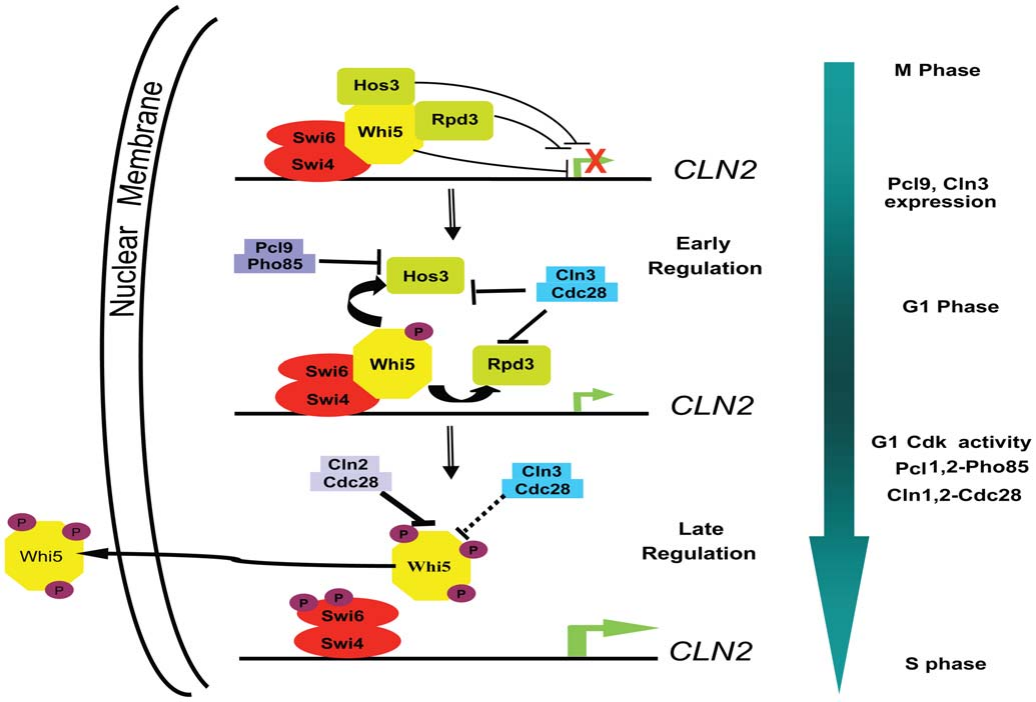
Model for CDK-dependent regulation of Whi5 activity and G1/S-specific transcription. Shown is a schematic of the disruption of interactions between Whi5 and the HDACs, Hos3 and Rpd3, by Cln3-Cdc28 and Pcl9-Pho85-dependent phosphorylation, leading to transcription of G1 genes, including the *CLN1* and *CLN2* cyclins. Whi5 is then further phosphorylated by Cln1- and Cln2-Cdc28 complexes leading to complete disassembly of the Whi5-SBF complex, Whi5 nuclear export and a burst in gene expression necessary for the G1/S phase transition.

On the basis of these and other observations, we propose that Whi5 functional regulation involves perturbation of specific HDAC-Whi5 interactions and requires the concerted activity of both Cdc28 and Pho85 (summarized in Figure 12). Interestingly, our genetic observations support a model whereby Pcl-Pho85 preferentially targets the Hos3-Whi5 interaction illustrating a functional distinction between the two CDKs. While Pho85 associates with several cyclin subunits, only Pcl9 exhibits temporal expression and localization patterns compatible with such a function. *PCL9* is expressed at the M/G1 phase transition and encodes a short-lived protein localized exclusively to the nucleus in early G1 phase [27],[60],[61]. Cln3 is also present in early G1 cells, but shows a complex localization pattern, with significant retention to the ER in early G1 cells, followed by chaperone-mediated release into the nucleus in late G1 phase [62]. How the specific features of Pcl9 and Cln3 localization might influence the timing of HDAC inhibition remains to be explored.

The second component of Whi5 regulation is predicated on previous studies indicating that G1/S gene expression is preceded by Whi5-SBF complex dissociation and subsequent nuclear export of Whi5 (Figure 12) [13]. Unlike early regulatory events, Cdc28 activity is both necessary and sufficient to drive these events since neither SBF binding to Whi5 nor nuclear localization of Whi5 was adversely affected in a *pho85*Δ mutant (Figure 7). Also, we are able to detect binding of SBF in vivo to *CLN2* promoters when *PHO85* is deleted (Figure 3C). However, both purified Cln3-Cdc28 and Pcl9-Pho85 failed to affect Whi5-SBF stability in vitro, while complex disruption was effectively achieved in the presence of Cln2-Cdc28 kinases (Figure 7). Cln3-Cdc28 and Pcl9-Pho85 may have a more pronounced effect on the Whi5-SBF complex in vivo. Alternatively, Cln3- and Pcl9-CDKs may act primarily as agonists of HDAC interactions while physical interactions with SBF and nuclear export are optimally mediated by the late G1 CDKs, Cln1- and Cln2-Cdc28. Indeed, recent work reveals activation of *CLN2* expression while Whi5 remains bound to the promoter (H. Wang, L.B. Carey, Y. Cai, H. Wijnen, and B. Futcher, personal communication). Such a mechanism may serve to sharpen the onset, as opposed to the timing, of G1/S gene expression thus ensuring a sustained transcriptional burst and irreversible commitment to cell division [13]. Consistent with this idea, recent analysis of cyclin gene expression using a single cell assay affirms that positive feedback involving the Cln1 and Cln2 cyclins induces the G1/S regulon, and that this regulatory feedback is important for maintaining coherence of gene expression at Start [63].

SBF promoter recruitment depends on a series of well-organized chromatin remodeling events [7],[36]. SBF, in turn, regulates the recruitment of the general transcription machinery via a two-step process beginning with the mediator complex followed by CDK-dependent recruitment of RNA PolII, TFIIB, and TFIIH [9]. Previous studies suggested that this CDK requirement stems from Whi5, which in its unphosphorylated state, remains bound to SBF and occludes the basal transcription machinery from binding specific promoters [13]. We have extended this model to include a role for HDAC activity. We predict that Hos3 and Rpd3 contribute to Whi5 repression by preventing holoenzyme access to chromatin. During states of high CDK activity, Cdc28 and Pho85 abrogate Whi5-HDAC and Whi5-SBF interactions and initiate transcription. Consistent with our model, Pcl9 and Cln3 cyclins localize to G1 promoters and Whi5 remains associated with G1-specific promoters in the absence of HDAC-promoter interactions (Figure 3; H. Wang, L.B. Carey, Y. Cai, H. Wijnen and B. Futcher, personal communication). However, Whi5 may also repress transcription by additional mechanisms since its activity is partially retained in *hos3*Δ *rpd3*Δ mutants (Figure 9).

Rpd3 is a well-characterized HDAC that accomplishes most of its functions as part of a large protein complex [37]. The Rpd3-Sin3 deacetylase complex has long been implicated as a cell cycle regulator required for silencing *HO* gene expression to prevent mating type switching in newly budded cells [64],[65]. Our observations that Whi5 associates with Rpd3 and our genetic data linking G1 Cdks, Whi5, and Rpd3 reveal a more general role for Rpd3 in G1/S-phase specific transcription. These data are consistent with observations from Futcher and colleagues that the Rpd3 protein can be detected at the *CLN2* promoter and that the amount of Rpd3 at the promoter is decreased when *CLN3* is induced (H. Wang, L.B. Carey, Y. Cai, H. Wijnen and B. Futcher, personal communication). The Rpd3-Sin3 HDAC has also been connected to G1 transcription factors through the interaction of Sin3 with Stb1, a Swi6-binding protein [66]–[68]. Both Stb1 and Sin3 are required for repression of G1 transcription early in G1 phase [68]. Unlike Rpd3, Hos3 is largely uncharacterized, although a recent study suggests a role for Hos3 in yeast apoptosis upon exposure to oxidative radicals [69]. We have uncovered an additional role for Hos3 in Whi5-mediated transcriptional repression.

A question that arises from our observations is what advantage does combinatorial kinase regulation impart on specific biological processes such as G1/S cell cycle progression? Contributions from multiple CDKs may provide the precision and accuracy necessary for rapid definitive decisions that irreversibly affect cellular fate. Indeed, distributive multisite phosphorylation mechanisms exhibit ultrasensitivity with respect to kinase concentration, thereby creating a “switch-like” behavior in biological circuits [70]. Since cell cycle transitions typically display switch-like attributes, multisite phosphorylation by various kinase combinations may prove to be a rule rather than the exception amongst CDK targets, including key cell cycle regulators such as Whi5. In fact, a recent computational analysis showed enrichment of multiple closely spaced consensus sites for Cdc28 substrates in yeast, a pattern that proved predictive of likely CDK targets [71].

Although kinase combinations are likely necessary for cell cycle regulation, the contribution of each individual kinase may vary depending on specific signals and environmental stimuli. In certain environments, Pcl-Pho85 may have more dramatic, condition-specific effects on Whi5 function than Cdc28 analogous, perhaps, to the regulation of Rb that is required for quiescence and prevention of apoptosis [72],[73]. Previous studies indicate that Whi5 localizes to nuclei in stationary phase cells suggesting that Whi5 may also play a role in G0 [13]. Interestingly, Pho85 is required for survival in starvation conditions and plays an important role during stationary phase [74]–[76]. Furthermore, CDK5, the mammalian Pho85 homolog, induces apoptosis in neuronal cells via Rb phosphorylation [77]. Whether Whi5 activity is more prominently affected by Pcl-Pho85 in response to stationary or stress conditions requires additional investigation.

Similarities between metazoan and yeast cell cycle regulation are increasingly evident as we continue to characterize Whi5 function. For example, similar to proposed Pcl9/Cln3 “early” phase regulation (Figure 12), cyclinD-CDK4/6 phosphorylates Rb to promote HDAC dissociation and E2F transcriptional activation. E2F activation then leads to cyclin E expression, which, similar to Cln1/2 “late” phase regulation (Figure 12), may establish a positive feedback loop whereby cyclinE-CDK2 activity disrupts Rb-promoter interactions and stimulates G1-transcription further [15]. Despite these similarities, the importance of multiple regulatory components in both yeast and mammalian systems remains poorly understood and may be most fruitfully dissected using the yeast model.

## Materials and Methods

### Yeast Strains, Growth Conditions, and Plasmids

The *S. cerevisiae* strains used are listed in Table 1. All gene disruptions and integrations were achieved by homologous recombination at their chromosomal loci by standard PCR-based methods and confirmed by PCR with flanking primers [78]. Standard methods and media were used for yeast growth and transformation. Two percent of galactose in the media was used to induce the expression of genes under the *GAL1* promoter. Synthetic minimal medium with appropriate amino acid supplements was used for cells containing plasmids. Appropriate amounts of 3-AT were added to SD-HIS plates to assess the expression of *HIS3* reporter genes. 10-fold serial dilutions (5–10 µl) of yeast cells were spotted onto plates with appropriate nutrition conditions to assess growth. Plasmids used in this study are listed in Table 2. In most cases, a DNA insert was amplified by PCR and inserted into a linearized vector by homologous recombination in yeast. Details of construction will be provided upon request.

**Table 1:** Yeast strains.

Strain	Genotype	Source or Reference
BY186	BY263 *MAT*a *swi4*Δ*HIS3*	[82]
BY263	*MAT*a *trp1 leu2 his3 ura3 lys2 ade2*	[29]
BY391	BY263 *MAT*a *pho85*Δ*LEU2*	[29]
BY451	BY263 *MAT*a *pcl2*Δ*LYS2*	[25]
BY462	*MAT*a *leu2 his3 ura3 cdc28-13*	M. Tyers
BY465	*MAT*a *leu2 his3 ura3 cdc28-4*	M. Tyers
BY490	BY263 *MAT*a *pho80*Δ*HIS3*	[25]
BY628	BY263 *MAT*a *pcl1*Δ*LEU2*	[25]
BY653	BY263 *MAT*a *cln3*Δ*URA3*	This study
BY694	BY263 *MAT*a *pcl9*Δ*HIS3*	[25]
BY760	BY263 *MAT*a *pcl1*Δ*LEU2 pcl9*Δ*HIS3*	[25]
BY764	BY263 *MAT*a *pcl1*Δ*LEU2 pcl2*Δ*LYS2 pcl9*Δ*HIS3*	[25]
BY867	BY263 *MAT*a *pho85*Δ*TRP1*	[25]
BY1446	BY263 *MAT*α *cln3*Δ*URA3 pho85*Δ*LEU2 whi5*Δ*KAN^R^*	This study
BY1502	Y2454 *MAT*α *pho85*Δ*LEU2*	[74]
BY2507	BY4741 *MAT*a *WHI5^myc^::KAN^R^*	M. Tyers
BY2948	BY4741 *MAT*a *cln3*Δ*HPH^R^ bck2*Δ*NAT^R^ pGAL-CLN3 URA3*	This study
BY4148	BY4741 *MAT*a *GALpr-HA-CDC20::KAN^R^ pho85*Δ*NAT^R^ PCL9^myc^*	This study
BY4151	BY4741 *MAT*a *GALpr-HA-CDC20::KAN^R^*	This study
BY4152	BY4741 *MAT*a *WHI5^myc^::KAN^R^ pho85*Δ*NAT^R^*	This study
BY4153	BY4741 *MAT*a *WHI5^myc^::KAN^R^ cdc28-4*	This study
BY4154	BY4741 *MAT*a *WHI5^myc^::KAN^R^ cdc28-4 pho85*Δ*NAT^R^*	This study
BY4242	BY4741 *MAT*a *GALpr-HA-CDC20::KAN^R^cln1*Δ*NAT^R^ cln2*Δ*HPH^R^*	This study
BY4269	BY4741 *MAT*α *GALpr-HA-CDC20::KAN^R^cln3*Δ*URA3 pho85*Δ*LEU2^R^*	This study
BY4270	BY4741 *MAT*α *GALpr-HA-CDC20::KAN^R^cln3*Δ*URA3 pho85*Δ*LEU2 whi5*Δ*KAN^R^*	This study
BY4273	BY4741 *MAT*a *GALpr-HA-CDC20::KAN^R^cln3*Δ*URA3*	This study
BY4274	BY4741 *MAT*a *GALpr-HA- CDC20::KAN^R^ pho85*Δ*LEU2*	This study
BY4288	BY4741 *MAT*a *WHI5^myc^::KAN^R^ cln3*Δ*LEU2*	This study
BY4289	BY4741 *MAT*a *WHI5^myc^::KAN^R^ cln1*Δ*NAT^R^ cln2*Δ*HPH^R^*	This study
BY4290	BY263 *MAT*a *cln3*Δ*TRP1*	This study
BY4292	BY263 *MAT*α *cln3*Δ*URA3 pho85*Δ*LEU2 whi5*Δ*KAN^R^*	This study
BY4293	BY263 *MAT*a *hos3*Δ*KAN^R^*	This study
BY4294	BY263 *MAT*a *rpd3*Δ*NAT^R^*	This study
BY4295	BY263 *MAT*a *hos3*Δ*KAN^R^ rpd3*Δ*NAT^R^*	This study
BY4296	BY263 *MAT*a *cln3*Δ*TRP1 hos3*Δ*KAN^R^*	This study
BY4297	BY263 *MAT*a *cln3*Δ*TRP1 rpd3*Δ*NAT^R^*	This study
BY4298	BY263 *MAT*a *cln3*Δ*TRP1 hos3*Δ*KAN^R^ rpd3*Δ*NAT^R^*	This study
BY4299	BY263 *MAT*a *pho85*Δ*LEU2 hos3*Δ*KAN^R^*	This study
BY4300	BY263 *MAT*a *pho85*Δ*LEU2 rpd3*Δ*NAT^R^*	This study
BY4301	BY263 *MAT*a *pho85*Δ*LEU2 hos3*Δ*KAN^R^ rpd3*Δ*NAT^R^*	This study
BY4302	BY4741 *MAT*a *ho*Δ*::SCB:HIS3::URA3*	This study
BY4303	BY4741 *MAT*a *ho*Δ*::SCB:HIS3::URA3 cln3*Δ*NAT^R^*	This study
BY4304	BY4741 *MAT*a *ho*Δ*::SCB:HIS3::URA3 pho85*Δ*LEU2*	This study
BY4305	BY4741 *MAT*a *ho*Δ*::SCB:HIS3::URA3 cln3*Δ*NAT^R^ pho85*Δ*LEU2*	This study
BY4306	BY4741 *MAT*a *ho*Δ*::SCB:HIS3::URA3 cln3*Δ*NAT^R^ pho85*Δ*LEU2 whi5*Δ*KAN^R^*	This study
BY4307	BY4741 *MAT*a *ho*Δ*::SCB:HIS3::URA3 cln3*Δ*NAT^R^ whi5*Δ*KAN^R^*	This study
BY4308	BY4741 *MAT*a *ho*Δ*::SCB:HIS3::URA3 pho85*Δ*LEU2 whi5*Δ*KAN^R^*	This study
BY4309	BY4741 *MAT*a *HOS1^TAP^::HIS3*	This study
BY4310	BY4741 *MAT*a *HOS2^TAP^::HIS3*	This study
BY4311	BY4741 *MAT*a *HOS3^TAP^::HIS3*	This study
BY4312	BY4741 *MAT*a *HDA1^TAP^::HIS3*	This study
BY4313	BY4741 *MAT*a *HDA2^TAP^::HIS3*	This study
BY4314	BY4741 *MAT*a *HDA3^TAP^::HIS3*	This study
BY4315	BY4741 *MAT*a *RPD3^TAP^::HIS3*	This study
BY4454	BY263 *MAT*a *whi5*Δ*KAN^R^*	This study
BY4455	BY263 *MAT*a *cln3*Δ*URA3 pho85*Δ*LEU2 hos1*Δ*HIS5*	This study
BY4456	BY263 *MAT*a *cln3*Δ*URA3 pho85*Δ*LEU2 hos2*Δ*HIS5*	This study
BY4457	BY263 *MAT*α *cln3*Δ*URA3 pho85*Δ*LEU2 hos3*Δ*NAT^R^*	This study
BY4458	BY263 *MAT*α *cln3*Δ*URA3 pho85*Δ*LEU2 rpd3*Δ*NAT^R^*	This study
BY4459	BY263 *MAT*a *cln3*Δ*URA3 pho85*Δ*LEU2 hda1*Δ*HIS5*	This study
BY4461	BY263 *MAT*a *cln3*Δ*URA3 pho85*Δ*LEU2 hos3*Δ*KAN^R^ rpd3*Δ*NAT^R^*	This study
BY4462	BY2948 *whi5*Δ*KAN^R^*	This study
BY4463	BY2948 *hos1*Δ*KAN^R^*	This study
BY4464	BY2948 *hos2*Δ*KAN^R^*	This study
BY4465	BY2948 *hos3*Δ*KAN^R^*	This study
BY4466	BY2948 *rpd3*Δ*HIS5*	This study
BY4467	BY2948 *hda1*Δ*HIS5*	This study
BY4468	BY2948 *hos3*Δ*KAN^R^ rpd3*Δ*HIS5*	This study
BY4541	BY263 *pho85*Δ*LEU2 whi5*Δ*KAN^R^*	This study
BY4542	BY263 *cln3*Δ*TRP1 whi5*Δ*KAN^R^*	This study
BY4741	*MAT*a *leu2*Δ*0 his3*Δ*1 ura3*Δ*0 met15*Δ*0*	—
Y2454	*MATα mfa1*Δ *MFApr-HIS3 can1*Δ *his3*Δ*1 leu2*Δ*0 lys2*Δ*0*	[83]

Of the wt strains used in this study, both BY263 and BY4741 are derived from S288C background. All the other strains are derived from these two strains. BY263 is an *ssd1-d* strain; BY4741 is an *SSD1-V* strain and is the parent strain for the yeast deletion consortium. Y2454 is congenic to BY4741 and is the parent for query strains used in synthetic genetic array (SGA) experiments.

**Table 2:** Plasmids used in this study.

Name	Relevant Genotype	Source
pEG-H	*pGAL1-GST URA3 2* µm	M. Snyder
pMT3164	*pGAL-c-FLAG LEU2 CEN*	Y. Ho
pMT3446	*GST-WHI5 in pGEX4T1* (*Escherichia coli* expression vector)	M. Tyers
pMT3586	*pGAL-WHI5-FLAG LEU2 CEN*	Y. Ho
pBA230v	*pGPD TRP1 2* µm	M. Funk
pBA330v	*pGPD LEU2 2* µm	M. Funk
pBA1820	*pGPD-HA-PCL1 LEU2 2* µm	This study
pBA1821	*pGPD-HA-PCL2 LEU2 2* µm	This study
pBA1822	*pGPD-HA-PCL9 LEU2 2* µm	This study
pBA1823	*pGPD-HA-PHO80 LEU2 2* µm	This study
pBA1973	*GST-WHI5 in pEG-H*	M. Snyder
pBA1974	*pGAL-PCL9-FLAG LEU2 CEN*	Y. Ho
pBA1975	*pMET-GST-WHI5 HIS3 CEN*	This study
pBA1976	*pGAL-8XLexAop-LacZ URA3 2* µm	This study
pBA1977	*pGPD-LexA TRP1 2* µm	This study
pBA1978	*pGPD-LexA-WHI5 TRP1 2* µm	This study
pBA1979	*pGPD-LexA-WHI5^12A^ TRP1 2* µm	This study
pBA1980	*pGPD-WHI5 TRP1 2* µm	This study
pBA1981	*pMET-WHI5-GFP HIS3 CEN*	This study
pBA2112	*pGAL-HA-PCL9 URA3 2* µm	J. Moffat
pBA2239	*GST-PCL1 in pGEX4T1* (*E.coli* expression vector)	This study
pBA2240	*GST-PCL2 in pAcGHLT* (baculovirus transfer vector)	This study
pBA2241	*GST-PCL9 in pAcGHLT* (baculovirus transfer vector)	This study
pBA2242	*GST-PHO80 in pAcGHLT* (baculovirus transfer vector)	This study
pBA2243	*GST-PHO85 in pAcGHLT* (baculovirus transfer vector)	This study
pBA2244	*GST-CLN2 in pAcGHLT* (baculovirus transfer vector)	This study
pBA2245	*GST-CLN3 in pAcGHLT* (baculovirus transfer vector)	This study
pBA2246	*GST-CDC28 in pAcGHLT* (baculovirus transfer vector)	This study
pBA2247	*pGAL-CLN2-FLAG LEU2 CEN*	Y. Ho
pBA2248	*pGAL-CLN3-FLAG LEU2 CEN*	Y. Ho
pBA2249	*pMET-GST-WHI5^12A^ HIS3 CEN*	This study

### Kinase Assays

The in vitro protein kinase assays monitored the incorporation of [^32^P] transferred from γ-^32^P-ATP to purified recombinant GST-Whi5. The reaction mixture for assays shown in Figure 2A contained 50 mM Tris-HCl (pH 7.5), 1 mM DTT, 10 mM MgCl_2_, and 1 µM ATP (including 20 µCi γ-^32^P-ATP) and 0.2 µg GST-Whi5 in 20 µl of total volume. 2 µl of a purified recombinant kinase (0.4 µg–0.8 µg) was added to the mixture and incubated at 30°C for 30 min. Purification of Cln and Pcl CDKs from insect cell expression systems have been previously described [13],[46]. Whi5 was then analyzed by SDS-PAGE and autoradiography. Kinase assays on immunoprecipitated proteins from yeast cell extracts were performed as described [13]. Kinase assays preceding the Whi5-SBF dissociation assay (Figure 7) were performed as described above except that 200 µM γ-^32^P-ATP was used instead of 1 µM. The final concentration of Cln3 and Pcl9 was 3 µM, and the final concentration of Cln2 was 60 nM (50-fold less).

### Quantitative β-Galactosidase Assays

Liquid β-galactosidase assays were performed as described [29]. Strains carrying appropriate plasmids were grown in synthetic minimal medium to mid-log phase, transferred to synthetic galactose medium, and incubated for 4 h. Cells were harvested and broken in lysis buffer (100 mM Tris-HCl [pH 8.0], 1 mM DTT, and 20% glycerol with protease inhibitors) with glass beads. The β-galactosidase activity was determined by adding 100 µl of total cell extract to 0.9 ml of Z buffer (100 mM Na_2_PO_4_, 40 mM NaHPO_4_, 10 mM KCl, 1 mM MgSO_4_, and 0.027% β-mercaptoethanol) and 200 µl ONPG (4 mg/ml) (Sigma). Units of β-galactosidase activity were determined as described [29].

### Whi5 Dissociation with SBF Complex In Vitro

The protein binding assay essentially followed the procedures described previously [13]. Briefly, 1 µl of insect cell lysate expressing SBF (Swi6-Swi4^FLAG^) was mixed with 1 µl of purified GST-Whi5 (∼0.1 µg) and 7 µl of M2 anti-FLAG resin (Sigma) in 8 µl of kinase buffer (50 mM Tris-HCl [pH 7.5], 1 mM DTT, and 10 mM MgCl_2_). The mixture was incubated at 4°C for 1 h with mixing. The beads bound to the SBF-Whi5 complex were then washed three times with kinase buffer, and mixed with various cyclin dependent kinases in kinase buffer with 0.2 mM ATP in a 20 µl volume. The kinase reaction was incubated at 30°C for 1 h. The soluble portion was taken out and mixed with 20 µl of 2×SDS-PAGE loading buffer. The beads in the tube were washed three times with kinase buffer before mixing with 15 µl of 2×SDS-PAGE loading buffer.

### Liquid Growth Assays

Strains containing galactose-inducible plasmids were grown to saturation in 2% raffinose media for 48 h. Expression of plasmids were induced by transferring into 2% raffinose 2% galactose media and liquid growth assays were performed as previously described over 36 h using a Tecan GENios microplate reader (Tecan) [79]. Average doubling (AveG) for each culture was calculated as previously described [79]. Growth rate for each mutant was calculated relative to the AvgG of the wt strain.

### Whi5-GFP Localization

The localization of Whi5-GFP was monitored in wt, *cdc28-4*, and *pho85*Δ strains. Cells expressing p*MET-GFP-WHI5* were grown to log phase in synthetic glucose medium without methionine. Cells were observed at a magnification of 1,000× using Nomarski optics and fluorescence microscopy and photographed by a Cascade 512B high-speed digital camera (Roeper Scientific) mounted on a Leica DM-LB microscope. Images were captured and analyzed by MetaMorph software (Universal Imaging Media).

### ChIP

The *pho85*Δ *PCL9^MYC^ GALpr-CDC20* and *pho85*Δ*whi5*Δ*PCL9^MYC^ GALpr-CDC20* cells were grown in YP-Galactose (YPG) medium to an optical density (OD_600_) of 0.4, blocked at M phase by growing in YPED medium for 3 h, and released into YPG medium. Samples were taken every 15 min after release and cross-linked with a final concentration of 1% formaldehyde. Wt and *swi4*Δ strains (for controls) were grown to OD_600_ of 0.6 in YPD. Formaldehyde cross-linking and preparation of whole-cell extracts were performed as previously described [80]. Immunoprecipitation were performed using 1∶200 dilution of α-myc monoclonal antibody (9E10), α-Swi6 or α-Swi4 polyclonal antibodies. The precipitates were washed twice with lysis buffer, once with LiCl detergent and once with Tris-buffered saline and processed for DNA purification. Enrichment at the *CLN2* promoter sequence was quantified with real-time PCR, using a dual fluorogenic reporter TaqMan assay in an ABI PRISM 7500HT Sequence Detection System as previously described [13].

### Other Materials and Methods

Recombinant GST-Pcl1 and GST-Whi5 were produced in a BL21 bacterial expression strain; other recombinant proteins were produced in insect cells infected with Baculovirus expression vectors [11],[46],[73]. Proteins were detected with 9E10 anti-Myc, 12C5 anti-HA, and M2 anti-FLAG monoclonal antibodies. FACS analysis of DNA content and cell size measurements were described previously [81].

## Supporting Information

Figure S1 **Expression levels of epitope-tagged Whi5 and Pcls.** Whi5 and Pcl abundance in the indicated strains was determined by immunoblotting. Cyclin proteins were detected using 12CA5 anti-HA antibodies whereas Whi5 protein was detected using anti-GST antibodies.
(0.93 MB EPS)

Table S1 **List of 53 synthetic lethal interactions involving** ***PHO85*** **that are not rescued by deletion of** ***WHI5*** **.**
(0.07 MB DOC)

## Abbreviations

3-AT: 3-aminotriazole
ATP: adenosine triphosphate
CDK: cyclin-dependent kinase
ChIP: chromatin immunoprecipitation
HDAC: histone deacetylase
Rb: retinoblastoma
Pcl: Pho85 cyclin
SDL: synthetic dosage lethality
wt: wild type
