# Supplementary material for: Dual Regulation by Pairs of Cyclin-Dependent Protein Kinases and Histone Deacetylases Controls G1 Transcription in Budding Yeast
Source: PLoS Biol. 2009 Sep 8;7(9):e1000188. doi: 10.1371/journal.pbio.1000188 (PMC2730531; doi:10.1371/journal.pbio.1000188)
Supplement: Table S1 — List of 53 synthetic lethal interactions involving PHO85 that are not rescued by deletion of WHI5 . (0.07 MB DOC) [file pbio.1000188.s002.doc]

**Table 1S** List of 53 synthetic lethal interactions involving *PHO85* that are not rescued by deletion of *WHI5*

| **Genes** | **Function category** |  |
| --- | --- | --- |
| ANP1 | Cell wall maintenance / regulation |  |
| MNN10 | Cell wall maintenance / regulation |  |
| HOC1 | Cell wall maintenance / regulation |  |
| VAN1 | Cell wall maintenance / regulation |  |
| PMR1 | Cell wall maintenance / regulation |  |
| FKS1 | Cell wall maintenance / regulation |  |
| SHC1 | Cell wall maintenance / regulation |  |
| ERD1 | Cell wall maintenance / regulation |  |
| WSC1 | Cell wall maintenance / regulation |  |
| WSC4 | Cell wall maintenance / regulation |  |
| ROM2 | Cell wall maintenance / regulation |  |
| TUS1 | Cell wall maintenance / regulation |  |
| RGD1 | Cell wall maintenance / regulation |  |
| BCK1 | Cell wall maintenance / regulation |  |
| SLT2 | Cell wall maintenance / regulation |  |
| SWI4 | Cell wall maintenance / regulation |  |
| CHO2 | Lipid / fatty acid metabolism |  |
| OPI3 | Lipid / fatty acid metabolism |  |
| SUR4 | Lipid / fatty acid metabolism |  |
| TAT2 | Membrane associated |  |
| SMF1 | Membrane associated |  |
| MCH4 | Membrane associated |  |
| BEM1 | Polarized growth |  |
| BEM2 | Polarized growth |  |
| BEM4 | Polarized growth |  |
| CLA4 | Polarized growth |  |
| BNI1 | Polarized growth |  |
| PAC10 | Polarized growth |  |
| YKE2 | Polarized growth |  |
| NUM1 | Polarized growth |  |
| TPM1 | Polarized growth |  |
| RTS1 | PP2A regulatory subunit |  |
| RTS3 | PP2A regulatory subunit |  |
| UME6 | Transcription |  |
| SRB9 | Transcription |  |
| SRB6 | Transcription |  |
| SFL1 | Transcription |  |
| SPT4 | Transcription |  |
| TPA1 | Transcription |  |
| GCR2 | Transcription |  |
| VPS29 | Vesicle trafficking |  |
| VPS35 | Vesicle trafficking |  |
| VPS38 | Vesicle trafficking |  |
| VPS44 | Vesicle trafficking |  |
| VAM3 | Vesicle trafficking |  |
| GSH1 | Other |  |
| BRE1 | Other |  |
| VIK1 | Other |  |
| YGL015C | Other |  |
| YJR142W | Other |  |
| YML122C | Other |  |
| LEM3 | Other |  |
|  |  |  |
